# New evidence for the role of gut microbiota on atrial fibrillation development

**DOI:** 10.1016/j.ebiom.2023.104622

**Published:** 2023-05-12

**Authors:** Christopher Papandreou

**Affiliations:** a Institute of Health Pere Virgili (IISPV), Reus, Spain; b Nutrition and Metabolic Health Research Group, Department of Biochemistry and Biotechnology, Rovira i Virgili University (URV), Reus, Spain; c Center of Environmental, Food and Toxicological Technology – TecnATox, Rovira i Virgili University, Reus, Spain

High-throughput sequencing technology has revolutionized gut microbiome research offering opportunities to study the impact of gut microbiota on host physiology. Significant interest has recently focused on the role of human gut microbiota in cardiovascular health. A growing body of evidence suggests that alterations in gut microbiota composition and disruptions in gut microbial metabolism are associated with CVD development.^1^

Atrial fibrillation (AF) is a heart rhythm disorder characterized by irregular and tremulous heart beats and is associated with significant CVD burden and high healthcare costs. Besides age and sex, a large number of cardiovascular risk factors may determine the manifestation and progression of AF such as hypertension, obesity, type 2 diabetes, dyslipidemia, coronary artery disease and heart failure. However, these CV risk factors partly explain (slightly >50%) the variability in AF occurrence and thus it is important to identify novel risk markers. Although the importance of gut microbiota on the progression of cardiovascular traits like atherosclerosis and classical CV risk factors has been investigated^2^ it remains unknown to which extend gut microbiota affects AF. In a previous small case–control study of 50 AF patients and 50 matched controls, Zuo K et al. observed that patients with AF exhibited significantly elevated richness and increased diversity of gut microbiota and perturbation in microbial composition which was correlated with dysregulated metabolic activity.^3^ Later, Zuo K et al. examined gut microbiota dysbiosis in AF types and found imbalances in microbial composition in patients with paroxysmal AF and persistent AF.^4^ More recently, Chen et al., found reduced alpha diversity in 53 AF patients, whereas the composition and abundance of their gut microbiota differed from that of healthy subjects and correlated with CV risk factors.^5^ Because of the growing interests in investigating the pathogenic role of gut dysbiosis in AF, large prospective cohort studies are needed to strengthen the level of evidence relative to the other observational study designs.

In this study published in *EBioMedicine*, Palmu et al.^6^ provides further observational evidence of the potential associations of gut microbiome and AF. Even though alpha and beta diversity indices did not discriminate participants with prevalent or incident AF from non-AF controls, the microbial composition was altered. Specifically, the authors found 9 and 8 genera associated with prevalent and incident AF, respectively. Interestingly, the majority of the genera linked with prevalent and incident AF have been previously shown to relate to hypertension in the FINRISK cohort^7^ and heart failure in a small case–control study^8^ suggesting that there may be shared underlying mechanisms. The authors also provided an external validation case–control study of 64 patients with AF and 74 controls and revealed that 6 out of 8 shared genera detected in both cohorts were shifted in the same direction. This is important, as assessing the generalizability of microbiota findings in other human populations is needed to justify scalability to broader investigations in the future.

This is the first large observational study with available microbiota data derived by whole genome untargeted shallow metagenomic sequencing, and adequate phenotypic and lifestyle evaluation, published to date, that examined the relationship between gut microbes and AF. Another strength of the study is the inclusion of an external validation study, making the findings more generalizable to the population. Furthermore, the study by Palmu et al.^6^ included in silico functional analysis to gain insights into the potential biological mechanisms underlying the association between gut microbiota and AF.

However, this study^6^ also has some limitations. While shallow shotgun sequencing can provide a more comprehensive view of the microbial community compared to amplicon-based sequencing methods such as 16S rRNA sequencing, its taxonomic resolution is still limited compared to deeper shotgun sequencing that covers more of the microbial genome. As a result, shallow shotgun sequencing may only be able to identify bacterial taxa up to the genus level, which may limit the ability to detect specific bacterial species that may be more strongly associated with AF, but still it represents a valuable tool for investigating the potential role of gut microbiota in the development of AF in large-scale epidemiological studies where cost and complexity are major concerns. Another limitation is that despite the in silico functional analysis, the lack of metabolites measurements in the study limited the ability to confirm the predicted functional changes and to establish causality between gut microbiota and AF.

While these limitations are significant, this large observational study provided a new direction of CV research by proposing a number of genera that differed in abundance in prevalent or incident AF from that in non-AF individuals. However, the causal relationship between gut microbiota and AF remains unclear. Recently, a bidirectional Mendelian randomization analysis using a Chinese cohort showed that AF had potential causal effects on the abundance of specific gut microbes.^9^ Given that microbiota may facilitate AF development through their bioactive metabolites measured in fecal and blood samples,^10^ further investigations to determine whether gut microbiota-mediated metabolic alterations would have arrhythmogenic effects accelerating the susceptibility to and progression of AF are needed to fully understand the biological function of microbiome. Whether modulating the intestinal microbiome and metabolism represents a promising approach to prevent AF requires further investigation.

## Declaration of interests

The author declares no conflicts of interest or disclosures in relation to the present commentary.
